# Structural Ceramic Nanocomposites: A Review of Properties and Powders’ Synthesis Methods

**DOI:** 10.3390/nano5020656

**Published:** 2015-04-28

**Authors:** Paola Palmero

**Affiliations:** Department of Applied Science and Technology, INSTM R.U. PoliTO, LINCE Lab., Politecnico di Torino, Corso Duca degli Abruzzi, 24, Torino 10129, Italy; E-Mail: paola.palmero@polito.it; Tel.: +39-011-090-4678; Fax: +39-011-090-4624

**Keywords:** ceramics, nanocomposites, powder synthesis, microstructure, properties

## Abstract

Ceramic nanocomposites are attracting growing interest, thanks to new processing methods enabling these materials to go from the research laboratory scale to the commercial level. Today, many different types of nanocomposite structures are proposed in the literature; however, to fully exploit their exceptional properties, a deep understanding of the materials’ behavior across length scales is necessary. In fact, knowing how the nanoscale structure influences the bulk properties enables the design of increasingly performing composite materials. A further key point is the ability of tailoring the desired nanostructured features in the sintered composites, a challenging issue requiring a careful control of all stages of manufacturing, from powder synthesis to sintering. This review is divided into four parts. In the first, classification and general issues of nanostructured ceramics are reported. The second provides basic structure–property relations, highlighting the grain-size dependence of the materials properties. The third describes the role of nanocrystalline second-phases on the mechanical properties of ordinary grain sized ceramics. Finally, the fourth part revises the mainly used synthesis routes to produce nanocomposite ceramic powders, underlining when possible the critical role of the synthesis method on the control of microstructure and properties of the sintered ceramics.

## 1. General Issues of Nanostructured and Nanocomposite Materials

Recent advances in the production of nanocrystalline ceramic powders with novel properties have stimulated the research to create multi-functional engineering materials by designing structures at the nanometric scale. Such recent enthusiasm in nanotechnology has also motivated the development of nanocomposite ceramics, which is one of the most rapidly evolving areas in composites research.

Nanotechnology can be broadly defined as the creation, processing, characterization and utilization of materials, devices and systems with dimensions of the order of 10–100 nm, exhibiting novel and significantly enhanced physical, chemical and biological properties, functions, phenomena and processes, due to their nano-scale size [1,2]. A nanomaterial has a typical grain size <100 nm, whereas ultrafine-grained materials are characterized by grain size <500 nm [3].

On the other hand, the term “nanocomposite” comprises multiphase materials, where at least one constituent phase has dimension of less than 100 nm [2].

The concept of structural ceramic nanocomposites was proposed by Niihara in 1991 and can be seen as the adoption of the nanocomposite approach for the microstructural tailoring of structural ceramic composites [4].

**Figure 1:**
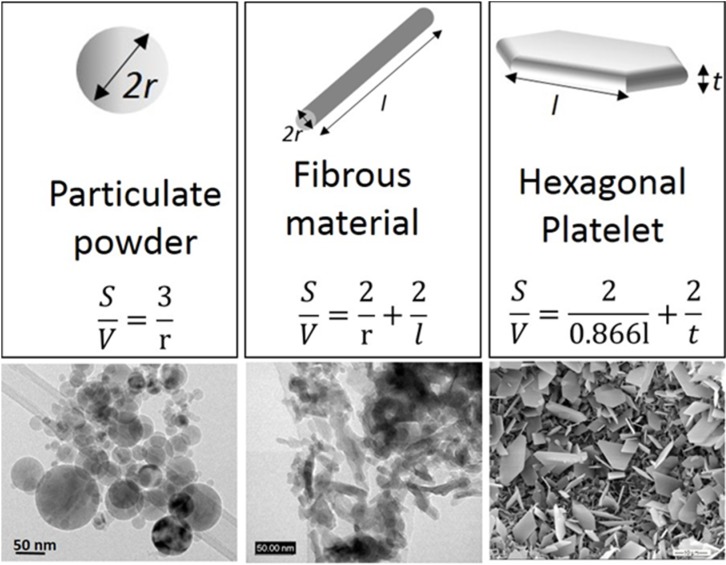
Schemes and images of different types of nanoreinforcements, redrafted from [2]. Surface area/volume relations for different reinforcement geometries are also displayed.

In order to classify the nanocomposite structures, we should firstly consider the different morphologies of the nanoscale reinforcements (Figure 1) [2]. In fact, nanoreinforcements can be grouped into three broad categories: (a) 3D-nanofillers (such as nanoparticles and nanospheres), which are relatively equiaxed with diameter <100 nm; (b) fiber or tube having diameter less than 100 nm and aspect ratio more than 100; (c) plate-like nanofillers, which can be layered materials with typically thickness of the order of 1 nm and aspect ratio in the other two directions at least of 25 [2].

The surface area/volume ratio for the different types of reinforcement is displayed in the same Figure. The change in particle diameter (2*r*), fibrous material diameter (2*r*) or layer thickness (*t*) from micrometer to nanometer changes the surface area/volume ratio by one or two orders of magnitudes (*i.e.*, from 1 μm to 10 or 100 nm) [2]. This means that with the drastic increase in interfacial area, the properties of the composite are more and more dominated by the properties of the interface or interphase.

**Figure 2:**
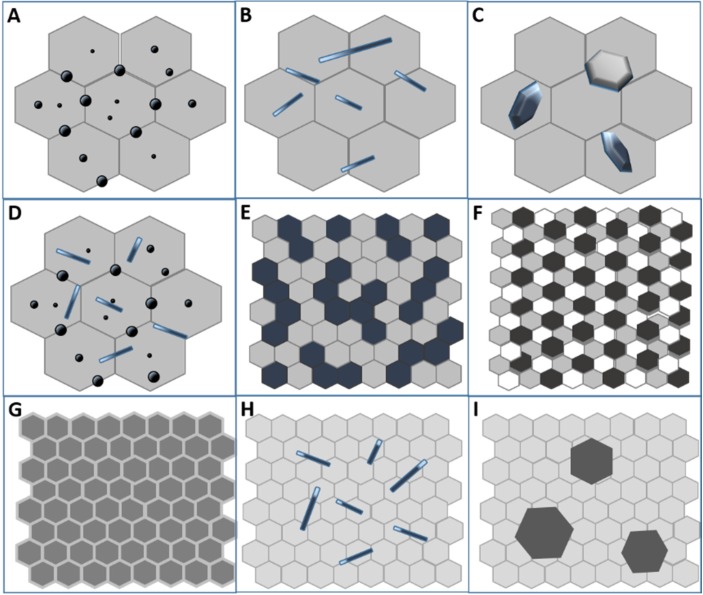
Scheme of common (nano)composite structures for ceramic materials, redrafted from [4] and [5]. (**a**) Micro/nano composite, with rounded nanoparticles occupying both inter- and intra-granular positions inside a micronic matrix; (**b**) Micro/nano composite, with elongated nanoreinforcements embedded in a micronic matrix; (**c**) Micro/nano composite, with platelet-like nanoreinforcements embedded in a micronic matrix; (**d**) Micro/nano composite, containing both rounded and elongated nanoreinforcements, embedded in a micronic matrix; (**e**) Bi-phasic composite made by two immiscible ultra-fine phases; (**f**) Multi-phasic composite made by three (or more) immiscible nanophases; (**g**) Nano/nanolayer type composite; (**h**) Nano- or micro-fibers embedded in a fine matrix; (**i**) Large second-phase precipitates embedded in a fine matrix.

Typical composite nanocrystalline bulk structures are schematically presented in Figure 2 [4,5].

The most common nanocomposite structures for ceramics consist of a micronic-sized matrix, in which nanoparticles are embedded [4]. In particular, the nanodispersions can be embedded in the matrix grains, located at the grain boundaries or occupy both inter- and intra-granular positions, as shown in Figure 2a. Examples of this structure can be found in the pioneer works of Niihara and co-workers related to nano-SiC particles dispersed in micronic Al_2_O_3_, S_3_N_4_ or Y_2_O_3_ matrix [6,7,8]. Other examples of micro/nano structures are displayed in Figure 2b,c, showing the presence of nanostructured elongated grains (whiskers, fibers or rod-like grains), (b) or platelets (c) embedded in a micronic matrix. The interest in such structures is explained by the *in situ* toughening effect exerted by the elongated grains and platelets, able to provide crack bridging and deflection mechanisms [9,10,11,12]. Recently, a new composite structure has been proposed in literature [13,14,15], consisting of both rounded and elongated nanostructured second-phase embedded in a conventional ceramic matrix, as shown in Figure 2d. Here, the rounded second-phase grains limit the matrix grain growth during sintering by exerting a *pinning* effect, with the aim of increasing the hardness and strength of the composites. The elongated grains, as previously explained, are chosen for their toughening effect, mainly due to crack deflection mechanisms. In Figure 2e, the structure of a nano/nano composite is displayed, consisting of approximately equiaxed nanoscale grains of two different phases. The two immiscible phases, contained in similar volume fractions, give rise to interconnected structures, in which each phase hinders the growth of the other, limiting the long-order interdiffusion. This concept can be extended to tri- or multiphasic systems, as shown in Figure 2f. In fact, raising the number of immiscible phases, the diffusion distance between homologous grains increases as well, thus further refining the overall composite microstructure [16]. In Figure 2g, a nanocrystalline composite consisting of grains of a single phase, divided by grain boundaries with a different chemical composition (second-phase) is represented [17]. Figure 2h,i show the structure of nanocrystalline composites consisting of elongated grains (h) or platelets (i) of one phase, embedded into a nanocrystalline matrix of the other phase.

**Figure 3:**
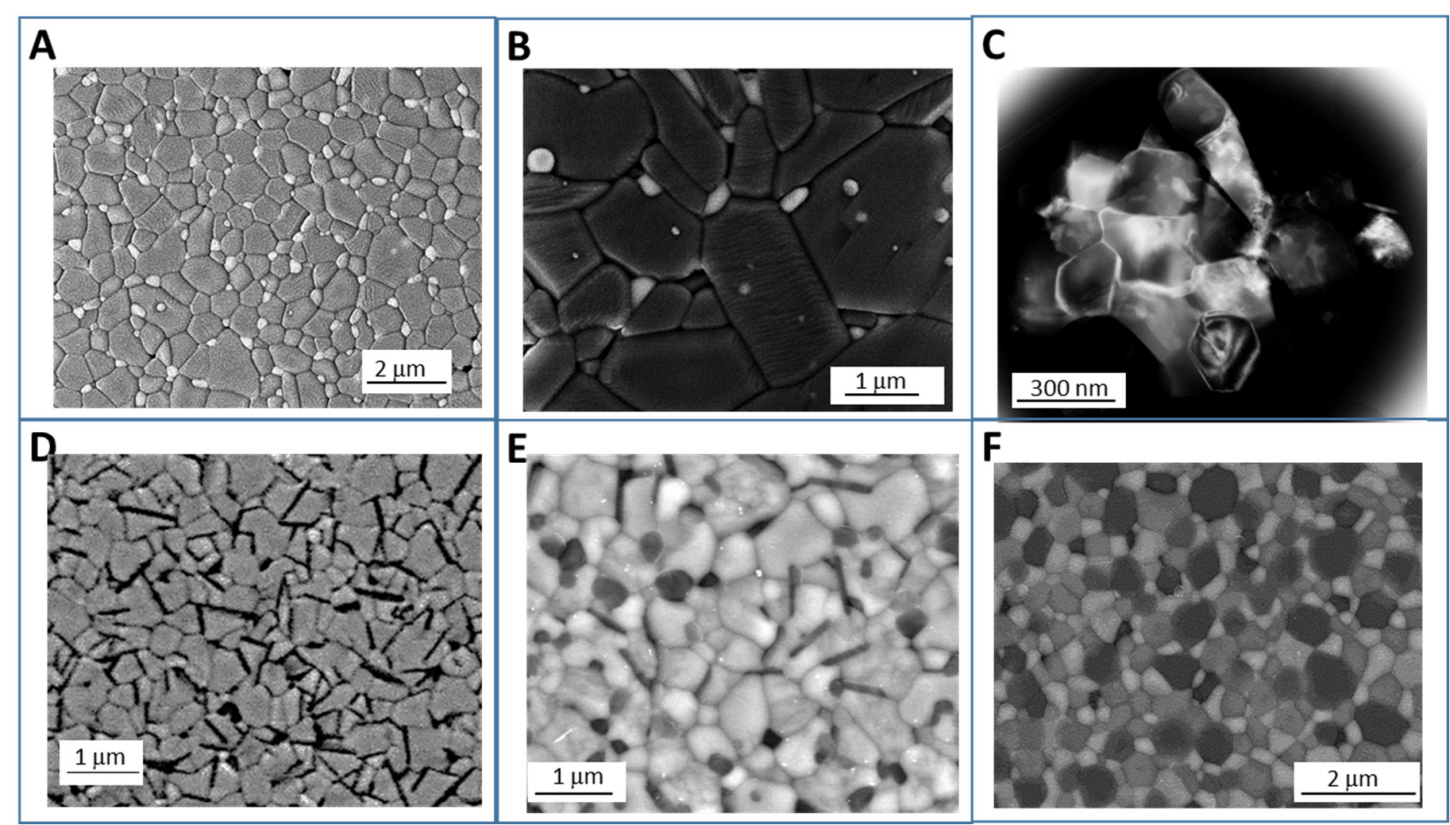
Example of different kinds of developed ceramic composite/nanocomposite structures. (**a**) Micro/nano Al_2_O_3_/Y_3_Al_5_O_12_ (YAG) composite, with YAG predominantly located at Al_2_O_3_ grain boundary [18]; (**b**) Al_2_O_3_/ZrO_2_ composites, in which ZrO_2_ grains occupy both inter and intragranular positions [18]; (**c**) Ultra-fine Al_2_O_3_/50_vol._%Y_3_Al_5_O_12_ (YAG) composite, with interpenetrating microstructure; (**d**) Y_2_O_3_-stabilized ZrO_2_ matrix containing elongated hexa-aluminate SrAl_12_O_19_ grains; (**e**) Triphasic composite, consisting on a ceria-zirconia matrix and containing both rounded α-Al_2_O grains and elongated hexa-aluminate SrAl_12_O_19_ grains [14]; (**f**) Ultra-fine Ultra-fine Al_2_O_3_/33_vol._%Y_3_Al_5_O_12_/33_vol._%ZrO_2_ composite [21].

In Figure 3, some examples of the above composite structures, experimentally developed, are reported [14,16,17,18,19,20,21,22,23].

In such different types of nanocomposite structures, a deep understanding of the materials’ behavior across length scales is required. Knowledge of how the nanoscale structure influences the bulk properties enables the design of the nanostructures, towards the fabrication of increasingly advanced and even multi-functional composites [2]. Once such knowledge is acquired, the tailoring of the desired composite structures is a further challenging step in the fabrication process. A successful approach requires the development of innovative concepts at each step of manufacturing, from the synthesis of composite nanopowders, to their processing and sintering. Nowadays, numerous technologies are available for producing highly pure, single-phase nanocrystalline powders [24]. In contrast, nanocomposite powders are still mainly produced by the traditional mixing and milling method, and it is striking the lack of advanced technologies applied to the synthesis of bi- or even multi-phasic systems, particularly at the industrial scale.

In this review, the attention focuses on ceramic-ceramic composite materials with macroscopically homogeneous structures, and in particular way will focus on particulate nanocomposite systems.

For a sake of completeness, this work will first consider the structural features of single-phase nanocrystalline ceramics (Section 2), and later those of nanostructured composites (Section 3). The final part (Section 4) is indeed dedicated to the synthesis methods of nanocomposite ceramic powders, with the aim of evidencing the key role of such manufacturing step in tailoring the micro/nanostructural features of the composites and the related final properties.

## 2. Why Nanostructured Ceramics?

Refining the material grain size in ceramics can induce significant changes on both mechanical and physical parameters. Therefore, the following paragraphs deepen some fundamental micro- or nano-structure-property relationships, with the aim of highlighting the size dependence of the materials properties.

### 2.1. Sintering and Grain Growth of Nanocrystalline Powders

To take advantage of the unique properties of bulk nanocrystalline materials, the nanometer range powders have to be densified into parts of certain properties, geometry and size. The key to the nanopowder consolidation process is to achieve densification with minimal microstructural coarsening and/or undesirable microstructural transformations. In particular, the grain growth normally occurring during the intermediate and final stages of sintering and the Ostwald ripening process should be avoided.

While the densification process for conventional powders is well known, the densification of nanopowders poses significant additional challenges, by both theoretical and practical points of view. First, due to the very large surface area of nanoparticles, agglomeration is an ubiquitous issue, affecting in a negative way the densification process. In addition, a still open question is whether the sintering mechanisms scale with grain size, and whether such mechanisms change when nanoscale is reached. A number of reviews have been already published addressing nanopowder processing and specific (nano)sintering issues [1,25,26,27].

As reviewed by Groza [25], nanopowders are highly unstable by a thermodynamic point of view. The tendency to reduce the excessively large surface area per unit volume of the nanocrystalline powders is the mechanism that drives the sintering process. The extra energy of a surface with a radius of curvature (*R*) may be calculated as a stress (σ), as given by the Laplace equation:

(1) $$\text{σ}=\frac{\text{γ}}{R}$$

where *γ* is the surface energy. In nanomaterials, this sintering stress may reach very high values. For instance, the sintering stress may be as large as 300 MPa in 10 nm particles compared to only 3 MPa for 1 μm particles, if γ has a typical value of 1.5 J/m^2^ [28].

From the kinetics point of view, we expect enhanced densification for processes that display a direct grain size dependence. For sintering, this dependence may be illustrated using the equation for the densification rate (dL/Ldt) developed by Johnson and co-workers, and used for all stages of sintering [29]:

(2) $$-\frac{dL}{Ldt}=\frac{\text{γΩ}}{kT}\;(\frac{\text{δ}D_{b}{\text{Γ}}_{b}}{d^{4}}+\frac{D_{\nu }{\text{Γ}}_{\nu }}{d^{3}})$$

where γ is the surface energy, Ω is the atomic volume, δ is the grain boundary width, *D_b_* and *D_v_* are the grain boundary and bulk diffusivities, Γ*_b_* and Γ*_v_* are functions of density, *k* is the Boltzmann constant, *T* the temperature and *d* is the grain size.

**Figure 4:**
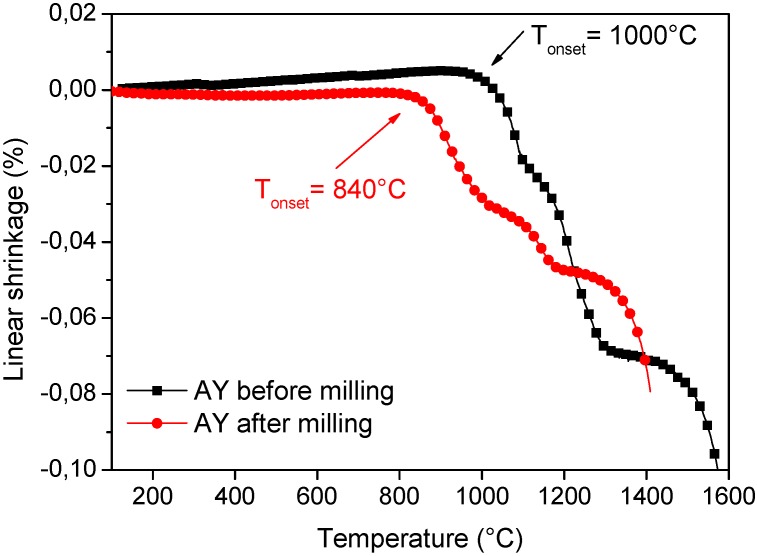
Linear shrinkage *versus* sintering temperature (during the heating step) of un-milled (black curve) and milled (red curves) AY powders. Maximum sintering temperatures were 1420 °C and 1600 °C for the milled and un-milled powders, respectively [34].

**Figure 5:**
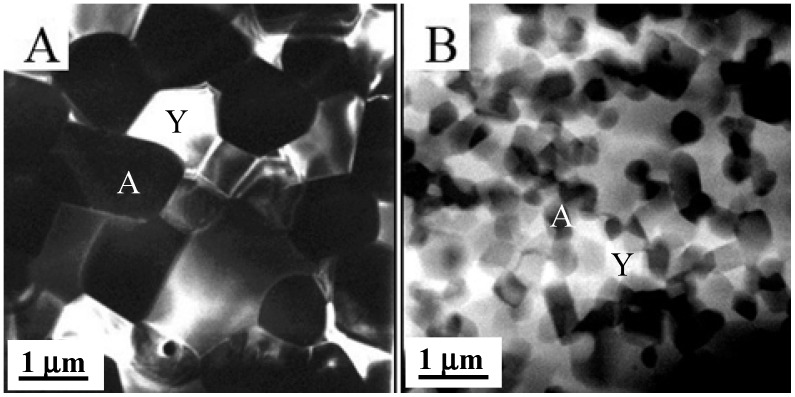
Transmission Electron Microscopy (TEM) images of AY materials. (**a**) Un-milled powder, sintered ad 1600 °C/3 h; (**b**) Milled powder sintered at 1420 °C/3 h. Characters A and Y refer to α-Al_2_O_3_ (black grains) and YAG (white grains), respectively.

From this equation, decreasing the grain size by one order of magnitude (e.g., from 1 μm to 100 nm) could enhance sintering rates by up to 4 orders of magnitude. Consequently, sintering of nanopowders may be accomplished at significantly lower temperatures and shorter time as respect to conventional powders. In fact, it was experimentally proved that the onset of sintering was significantly lower in nanoceramics (in the range 0.2–0.4 *T_m_*) compared to conventional materials (0.5–0.8 *T_m_*) [25,30,31,32,33]. However, to fully exploit the exceptional features of the nanocrystalline powders, they should be loosely dispersed, free from agglomerates. An example is provided in Figure 4, showing the sintering curves of Al_2_O_3_/50_vol._%Y_3_Al_5_O_12_ (YAG) powders (referred to as AY). The powder, synthesized by co-precipitation route and calcined at 900 °C to crystallize γ-Al_2_O_3_ and YAG phases, is made by nanocrystalline particles, with both phases having a size in the range of 20–50 nm [34]. However, the powder presents a significant agglomeration, being the agglomerates of about 3 μm in size. Extensive ball milling was necessary in order to reduce the agglomerate size to about 0.5 μm. The black and red curves represent the linear shrinkage curves *versus* sintering temperature of the powder before and after milling, respectively. We can observe that the dispersed powder presents an onset sintering temperature (about 840 °C) well lower than the un-milled one (about 1000 °C). Therefore, the maximum sintering temperatures were observed at 1420 °C for the milled material, and at 1600 °C for the un-milled one. After the heating step at these two different temperatures, the materials reached the same densification degree, which was about 50% of their theoretical density. Full densification was reached, in both cases, by performing an isothermal step of three hours at the maximum sintering temperatures. More details about the sintering behavior of the two materials can be found elsewhere [34]. The lowering of the maximum sintering temperature of the milled-AY material had a critical effect on its sintered microstructure. As we can see in Figure 5, the un-milled material (sintered at 1600 °C/3 h, Figure 5a) presented a very homogeneous but micronic microstructure; on the opposite, the milled material (sintered at 1420 °C/3 h, Figure 5b) showed an ultra-fine grain size (about 300 nm) for both Al_2_O_3_ and YAG phases.

In 1950, Herring [35] developed the classical scaling rule, correlating the effect of the particle size on the sintering time. The time, *t*, to achieve the same sintering condition is correlated to the powder particle size, *d*, by the equation:

(3) $$\frac{t^{1}}{t^{2}}={\left(\frac{d_{1}}{d_{2}}\right)}^{n}$$

where the exponent *n* depends on the sintering mechanism (in this equation, it is assumed the same sintering mechanisms, independently from the particle size). Considering the Arrhenius expression for temperature, the sintering temperature dependence on the particle size becomes:

(4) $$nln\left(\frac{d_{1}}{d_{2}}\right)=\frac{Q}{R[\left(\frac{1}{T_{1}}\right)-\left(\frac{1}{T_{2}}\right)]}$$

where *Q* is the activation energy for the predominant sintering mechanism, *R* is the gas constant, *d*_1_ and *d*_2_ are the different powder particle sizes and *T*_1_ and *T*_2_ are their respective sintering temperatures.

Considering Herring’s law, the sintering temperature dependence on the particle size may be calculated. Reasonable agreement of experimental and calculated temperatures was found in TiO_2_, and Al_2_O_3_ assuming certain diffusion mechanisms [25]. Zeng *et al*. [36] demonstrated that the Herring scale law can be used to predict the approximate sintering temperature of α-Al_2_O_3_ powder and showed that if the particles size was reduced to <20 nm, sintering below 1000 °C may be possible.

Yttria-stabilized zirconia powders (at different yttria contents) were submitted to a two-step sintering process, with the aim of achieving full densification but minimizing grain growth [37]. Pressureless two-step sintering is a relatively new method to obtain dense ceramics with restricted grain growth. As originally proposed by Chen and Wang in [38], the suppression of the grain growth in the final sintering stage may be achieved by exploring the kinetic window between grain boundary diffusion and grain boundary migration. A typical two-step sintering schedule consists of a first heating step to a peak temperature (*T_p_*) sufficient to reach a relative density higher than 75%. This step is then followed by a rapid cooling down to an isothermal plateau at a lower temperature (*T_d_*) for a relatively long time (*t_d_*), in which full densification should occur *via* a relatively slow grain boundary diffusion process. For yttria-stabilized zirconia, the conditions required for the effective two-step sintering were evaluated by shrinkage experiments performed on heating at constant rate. Data were interpreted on assuming the classical Herring’s law describing the shrinkage during the second stage of sintering [38]. By using this approach, high density (around 95%) tetragonal (ZrO_2_)_0.97_(Y_2_O_3_)_0.03_ and cubic (ZrO_2_)_0.92_(Y_2_O_3_)_0.08_ ceramics were obtained, respectively showing a nanometric and sub-micrometric microstructure.

### 2.2. Mechanical Behavior of Nanocrystalline Ceramics

#### 2.2.1. Hardness and Strength

Mechanical behavior of nanocrystalline materials has been the theme of over 500 publications and several review articles [24,25,26,39,40]. Most of these articles conclude that the yield stress (τ) and the microhardness (*H_v_*) of nanocrystalline materials can be 2–10 times higher than the corresponding coarse-grained polycrystalline materials, with the same chemical composition. These behaviors are described by the empirical Hall–Petch relationships, according to the following equations:

*τ* = *τ*_0_ + *k·d*^½^ (5)

*H_v_*=*H*_0_ + *k·d*^½^ (6)

where *H*_0_ is the intrinsic hardness dependent on frictional lattice resistance to dislocation motion [41], τ_0_ is a material constant [39], *k* is the material-specific strengthening coefficient, and *d* is the average grain size.

**Figure 6:**
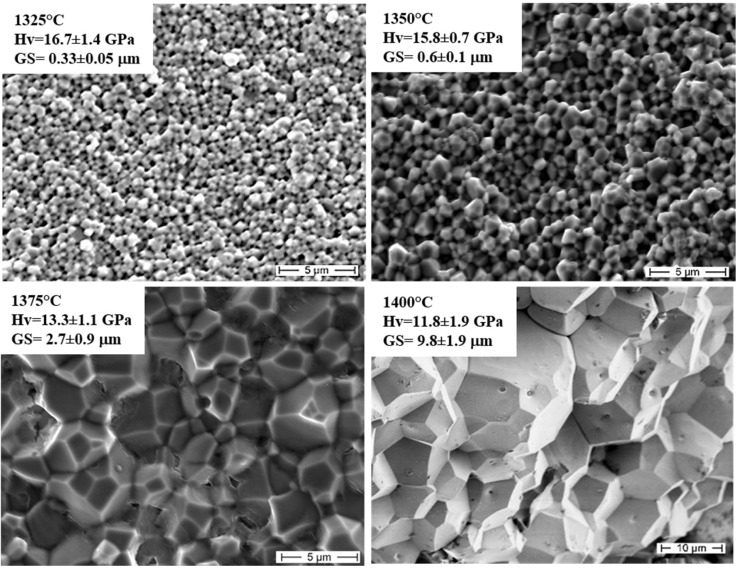
Field Emission Scanning Electron Microscopy (FESEM) micrographs of Y_3_Al_5_O_12_ (YAG) sintered by Spark Plasma Sintering (SPS) at increasing temperatures (Hv = average Vickers hardness; GS = average grain size) [41].

The Hall-Petch law is based on the theoretical [40] and experimental [42] observation that grain boundaries impede dislocation slip, which is the principal deformation mechanism accommodating strain during plasticity. In addition, it was proved that smaller grains limit the size of dislocation pile-up, which affects how easily dislocations can traverse grain boundaries and travel from grain to grain. Therefore, a higher applied stress is necessary to propagate dislocations from grain to grain and to induce a permanent deformation to a material: as a result, both yield strength and hardness are enhanced [39]. As an example, Figure 6 displays different microstructures of YAG ceramics, fabricated at increasing Spark Plasma Sintering (SPS) temperatures (from 1325 °C to 1400 °C), reaching full density [43]. Average grain size and Vickers hardness were determined for all materials. As shown in Figure 6, an inverse proportion between grain size and hardness can be clearly stated [44].

However, further refinement of grain size may lead to lower yield stress. Thus, some materials exhibited the so-called “inverse” Hall-Petch behavior, showing a decreased hardness with further reduction of grain size. While this phenomenon has been widely explored in nanocrystalline metallic materials, few data refer to nanocrystalline ceramics due to the difficulty associated to the fabrication of high-quality, dense nanostructured materials. In spite of this, a recent paper of Wollmershauser *et al*. [45] showed that the hardness of magnesium aluminate spinel, used as a model hard ceramic material, rigorously followed the Hall-Petch relationship down to grain sizes of 28 nm.

Besides the Hall-Petch equations, the Griffith theory also provides fundamental understanding of the mechanical behavior of ceramics. In fact, for elastic brittle materials, the fracture behavior can be analyzed by using the Griffith equation:

(7) $${\text{σ}}_{f}=\;\frac{K_{IC}}{Y\;\sqrt{\text{π}a}}$$

where σ*_f_* is the strength, *K_IC_* is the fracture toughness, *a* is the flaw size and *Y* is a geometric factor related to the failure origin, approximately equal to 1 [46]. From this equation, we can see that for increasing the strength of a brittle material, both an increase of *K_IC_* and a decrease of *a* are needed. Flaws of some statistically distributed size are always present in a ceramic material. They may arise during manufacturing or in subsequent handling of the material. Since polycrystalline ceramics are usually manufactured by sintering a powder compact at high temperature, the flaws may arise from non-uniformities in the particle packing. The refinement of the microstructure allows decreasing the material flaw size produced during processing: for this reason, it is generally assumed that the fracture strength in nanoceramics is higher than in conventional micronic-sized materials.

#### 2.2.2. High-Temperature Mechanical Properties

A further difference between nanocrystalline and coarse-grained materials lies in the high-temperature mechanical behavior. The creep behavior of coarse-grained materials has been extensively studied over the past four decades. These studies allowed establishing that the creep behavior of a polycrystalline material can be represented by the following expression [47]:

(8) $$\dot{\text{γ}}=A(\frac{DGb}{kT}){\left(\frac{b}{d}\right)}^{s}{\left(\frac{\text{τ}}{g}\right)}^{n}$$

with:

(9) $$D=D_{0}\exp(-\frac{Q}{RT})$$

where γ is the shear creep rate, *A* is a dimensionless constant, *D* is the diffusion coefficient that characterizes the creep process, *G* is the shear modulus, *b* is the Burgers vector, *k* is Boltzmann’s constant, *T* is the absolute temperature, *d* is the grain size, *s* is the grain size sensitivity, τ is the applied shear stress, *n* is the stress exponent, *Q* is the activation energy for the diffusion process that controls the creep behavior, *D*_0_ is the frequency factor for diffusion.

Starting from these general equations, two models have been formulated: the former, the Nabarro–Herring creep [48,49] involving the diffusion of vacancies through the grain volume; the latter, the Coble creep [50] involving the diffusion of vacancies along the grain boundaries. Once again, there is a role of the grain size on these behaviors: in fact, it was reported [51] that Coble mechanism could prevail over the Nabarro-Herring one at very small normalized grain size. Particularly, the Coble creep is defined by the following equation:

(10) $${\dot{\varepsilon }}_{Co}=\;A_{Co}\;\frac{Gb\;D_{gb}}{kT}\;{\left(\frac{b}{d}\right)}^{3}\;\frac{\text{σ}}{G}$$

showing a cubic dependence of the creep rate on *d.* A reduction in grain size from 10 μm to 10 nm would produce an increase in the creep rate by nine orders of magnitude, all else being constant, thus suggesting the possibility of deformation at room temperature [52,53]. In nanocrystalline ceramics, this feature has not been demonstrated yet. A previous study on nano-scale TiO_2_ has shown extensive ductility at room temperature [54]; however, subsequent investigations showed that ductility was due to the presence of porosity, an extrinsic effect not directly related to grain size [55,56,57]. This example clearly shows that the nanocrystalline domain of ceramic materials deserve further investigations. In fact, contrary to metals and alloys, few and sometime contradictory data refer to ceramic materials, as the fabrication of highly dense, nanostructured ceramics is still a complex issue.

## 3. Why Nanocomposites Ceramics?

### 3.1. Sintering and Grain Growth of Nanocomposite Ceramics

The addition of nanocrystalline second-phases to ordinary grain sized ceramics is very effective in reducing the particles grain growth during sintering. In fact, dopants or second-phase particles, which are insoluble in the matrix phase, can decrease the coarsening kinetics of the matrix itself by reducing the grain boundary mobility through a particle *pinning* effect.

This behavior was described in the Zener’s theory [58] in 1948, showing that the inclusion exerts a drug force on the grain boundary given by:

*F*= *2*π*r* γsinθ cosθ (11)

where *F* is the pinning force, *r* is the particle radius and γ is the average boundary energy of the system. If particles are assumed to be spherical and γ to be isotropic, the maximum value of the force, *F_max_*, is given when θ = π/4:

*F_max_* = π*r*γ (12)

The number of particles per area of boundary, *N_s_*, can be approximated by considering the particles to be distributed randomly on the boundary. Therefore:

(13) $$N_{s}=\frac{3f}{4{\text{π}}^{2}}$$

where *f* is the volume fraction of particles. Combining Equations (12) and (13), the pinning stress can be derived:

(14) $${\text{σ}}_{z}=\frac{3f\text{γ}}{4r}$$

In this theory, we should consider that numerous assumptions have been made: for instance, it applies to spherical and randomly distributed particles; it is assumed that each particle in contact with the boundary exerts the maximum force, whereas the pinning force exerted by each particle also depends on the position of the boundary.

The force on a boundary due to non-spherical particles has been calculated in 1983 by Ryum *et al.* [59], who considered needle- and plate-shaped particles. They evaluated the force as a function of aspect ratio of the angle between the boundary and the axis of the particle. If was found that the pinning force is a function of particle shape, large forces being associated with large differentials in the cross sectional area presented to the boundary. Thus, a needle particle with an aspect ratio of 5 can exert a force of 2*F_max_* on a boundary. Since the pinning force differs from the spherical case, we might expect that the pinning stress would be similarly affected. However, if the particles are randomly orientated, the pinning stress given by the previous equation is still a good approximation.

Finally, for spherical particles, the dependence of the matrix grain size (*R*) on the particle size and fraction can be also estimated, according to the following relationship:

(15) $$R=\frac{4r}{3f}$$

**Figure 7:**
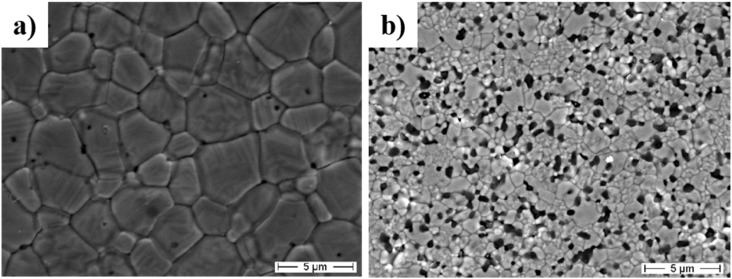
FESEM micrographs of (**a**) pure CeO_2_-stabilized ZrO_2_ and (**b**) the respective composite containing 16_vol._% of α-Al_2_O_3_, both sintered at 1450 °C for 1h [70].

The incorporation of nanoparticles into ceramics to control grain size during thermomechanical processing is now standard industrial practice. Second-phases such as fine YAG [60,61], ZrO_2_ [62,63,64,65], SiC [66], Si_3_N_4_ [67] NbC [68] are commonly added to Al_2_O_3_, to inhibit the matrix grain size and therefore increase the mechanical properties [69]. Figure 7 compares the microstructures of a pure CeO_2_-stabilized ZrO_2_ and the respective composite material, containing 16_vol._% of α-Al_2_O_3_, both sintered at 1450 °C for 1h. The fine and homogeneous distribution of the ultra-fine α-Al_2_O_3_ grains induces a very effective pinning effect on the ZrO_2_ grain boundaries: as a result, the ZrO_2_ average grain size decreases from about 4 μm in the monolithic material to about 0.7 μm in the composite [70].

### 3.2. Mechanical Properties of Nanocomposite Ceramics

#### 3.2.1. Hardness and Strength

As previously reported, the incorporation of ultra-fine second-phase particles can reduce the matrix grain growth. This has a general positive effect on the mechanical properties, since an increase in strength and hardness is expected on the ground of the Hall-Petch relation (see Equations (5) and (6)).

**Figure 8:**
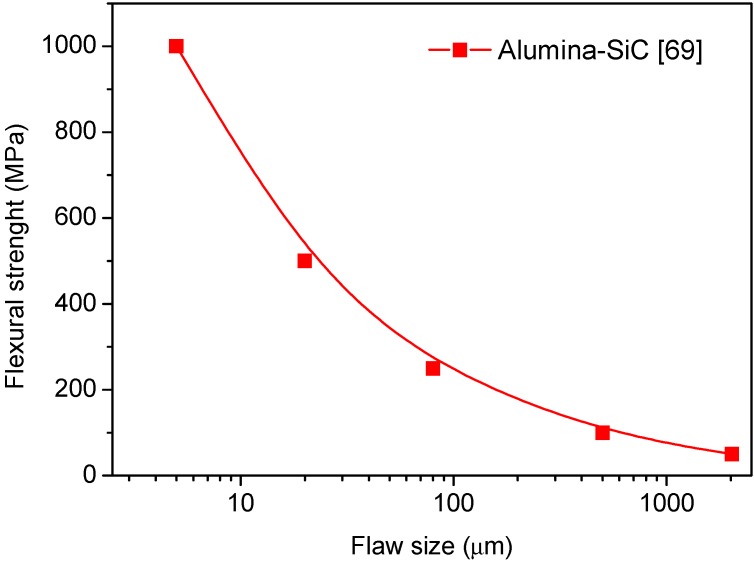
Evolution of flexural strength with the flaw size in Al_2_O_3_/SiC composites [69].

A further explanation of the increased strength of the nanocomposites is the reduction in the size of processing flaws. An example is provided by the Al_2_O_3_/SiC nanocomposite system, processed by extensive milling of Al_2_O_3_ and SiC raw powders. It was proved that during this step, the SiC particles act as grinding medium during attritor or ball milling and successfully destroy the Al_2_O_3_ agglomerates, which commonly cause large processing flaws [71,72]. This is illustrated in Figure 8 [72], in which we can see a continuous decrease of the flexural strength by increasing the flaw size in Al_2_O_3_/SiC ceramics. Values show that the flaw size should be very small to produce high-strength ceramics, meaning that a careful manufacturing during the ceramic processing is needed.

On the other hand, the SiC grains should also achieve an optimal distribution in the Al_2_O_3_ matrix, avoiding agglomeration. This is usually achieved for low SiC contents (up to 5_vol._%) allowing to increase the Al_2_O_3_ strength from about 350 MPa to about 1050 MPa, whereas further addition of SiC lowers the strength due to agglomeration phenomena [71].

In general, the strength of the composites can be estimated by the rule of mixtures. However, mechanisms that lead to a toughness change, such as microcracking or phase transformation (discussed later) may significantly alter the strength. In the previous example of Al_2_O_3_/SiC composite, the decreased strength above a certain volume fraction of SiC has been also imputed to microcrack linkage [73]. Similar results were obtained for the Al_2_O_3_/ZrC system: the flexural strength increased from about 300 to 550 MPa upon addition of 5–10_mol._% ZrC, above which it decreased to the initial value [74].

Ohji *et al*. [75] have proposed a “nanotoughening” effect for explaining the strengthening of the Al_2_O_3_/SiC system. A mechanism based on near-tip crack bridging by the nanosized SiC inclusions was considered to produce a modest toughness increment but a very steep *R*-curve, and this latter produces the very high fracture strength of the ceramic nanocomposites.

A further difference between monolithic ceramics and related nanocomposites is the fracture mode. Again with reference to the Al_2_O_3_/SiC system, it was observed that the fracture mode changed from a mixed inter/transgranular fracture mode in monolithic Al_2_O_3_ to pure transgranular mode in the composites, highlighting a grain boundary strengthening in this latter case. Ferroni and Pezzotti [76] estimated that the amount of transgranular fracture mode in monolithic Al_2_O_3_ was about 10%. This value increased to about 55%–85% in Al_2_O_3_/SiC nanocomposites, depending on the SiC amount. In particular, at the lowest SiC content (5_vol._%), the highest fraction of transgranular fracture mode was determined. However, this behavior was directly related to the amount of SiC particles located in inter or transgranular position as respect to the Al_2_O_3_ matrix. The predominant transgranular crack path was observed when SiC grains were mainly located within the Al_2_O_3_ matrix [76].

**Figure 9:**
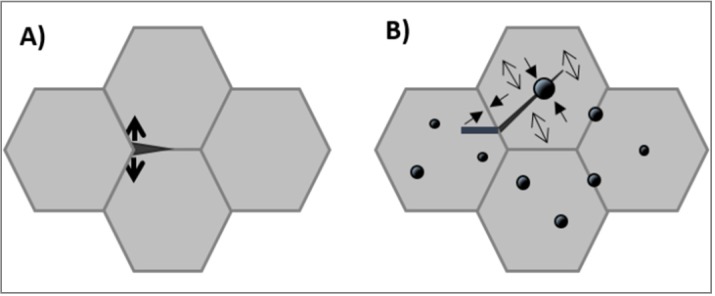
Schematic illustration of the residual stress field in (**a**) pure Al_2_O_3_, due to thermal expansion anisotropy during cooling and (**b**) Al_2_O_3_/SiC nanocomposites, due to thermal expansion coefficient mismatch between matrix and second-phase. In both figures, the preferential crack path is illustrated.

Conceivable reason for the grain boundary strengthening is the local internal stress, as for instance reported in [75,77]. In monolithic Al_2_O_3_, residual thermal stress is imputed to the thermal expansion anisotropy experienced by non-cubic ceramics during cooling from the sintering temperature. In these ceramics, triple points are inherently weak zones due to high residual stress during cooling. Figure 9*a* schematically shows a grain boundary microcrack due to local tensile stress, arising from thermal expansion anisotropy [76]. In composites, residual thermal stresses are imputed to the difference in thermal expansion coefficients between matrix (α*_m_*) and particle (α*_p_*), which cause strain during cooling from high-temperature sintering [71]. Thus, when second-phase grains, with lower thermal expansion, are embedded in the matrix grains, a hydrostatic compressive stress field can be generated on a grain boundary during cooling (see Figure 9b).

The thermal expansion misfit stress, σ_T_, inside a single spherical inclusion in an infinite matrix is described by the Selsing expression [77]:

(16) $${\text{σ}}_{T}=\frac{\langle {\text{α}}^{*}\rangle }{\frac{1+{\text{ν}}_{m}}{2E_{m}}+\frac{1-2{\text{ν}}_{p}}{E_{p}}}$$

where *<*α^*^> is the thermal expansion misfit strain, *E* and ν are Young’s modulus and Poissons’s ratio of the matrix (*m*) and particles (*p*). In the case in which α*_m_* > α*_p_*, the average thermal stress are compressive inside the second-phase particles and tensile in the matrix.

This is the case of the Al_2_O_3_/SiC system, where the SiC particles have lower thermal expansion coefficients than Al_2_O_3_. This induces compressive and tensile residual stress in the radial and tangential directions, respectively, of the Al_2_O_3_ matrix around the particle [76], as shown in Figure 9b. We can see that both Al_2_O_3_ and Al_2_O_3_/SiC grain boundaries are subjected to a hydrostatic compressive stress field, whereas the matrix is under a tensile stress. The direct consequence is the transgranular fracture mode, propagating inside both Al_2_O_3_ matrix and second-phase particles, as schematically illustrated in Figure 9b.

Finally, it should be mentioned that large differences in α coefficients between matrix and second phases (as in the case of Al_2_O_3_/SiC system) usually increase the possibility of microcracking, since the particles are under high local stresses. This limits the second-phase content in the ceramic matrix. A different case is provided by the Al_2_O_3_/TiC composite, which is a well-established cutting tool material, showing improved strength as compared to Al_2_O_3_ alone. In this system, high strength values can be retained at relatively high TiC contents, due to the smaller difference in α coefficients between TiC and Al_2_O_3_ [78], if compared to the Al_2_O_3_/SiC system.

#### 3.2.2. Fracture Toughness

Beside their strengthening effect, nano-sized second-phase particles can also increase the toughness of the monolithic ceramic material.

Toughness is a term used to define the resistance of a material to crack formation and propagation. It is also defined as the ability of a material to absorb energy before failure. Fracture toughness (*K_IC_*) is defined as the critical stress intensity above which a crack will propagate and result in failure of the material [73]. The subscript I represents mode I displacement, *i.e*., the load is perpendicular to the crack. For brittle materials, and according to Griffith’s equation, *K_IC_* can be expressed by rewriting Equation (7) as follows:

*K_IC_* = σ*_f_Y*√π*a* (17)

Being σ*_f_* the critical stress, *a* the half crack length, *Y* a constant depending on crack configuration and loading geometry.

**Figure 10:**
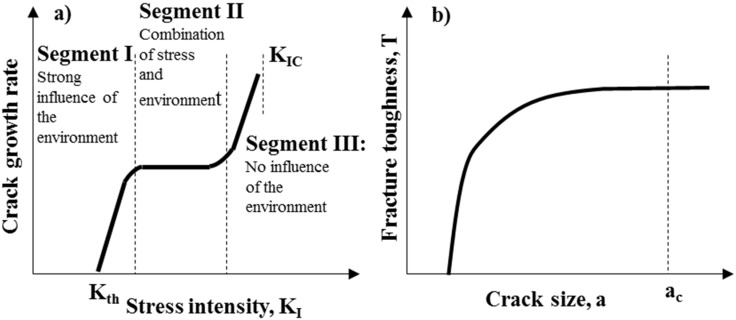
(**a**) Scheme of crack growth rate-stress intensity behavior; (**b**) Rising toughness with increasing crack size (*R*-curve behavior).

In ideally elastic (brittle) materials, crack propagation occurs catastrophically if the crack size is above a critical size, *a_c_*. At crack size *a* < *a_c_*, subcritical crack growth may occur due to environmental effects, *i.e*., stress corrosion, resulting in crack growth to the critical size and failure. The environmental effect on crack growth is best represented by crack velocity (*V*)-toughness (*K_I_*) curve [79], as shown in Figure 10a. Segment I corresponds to chemically enhanced crack growth: below the threshold value, *K_th_*, cracks do not propagate. Segment II (plateau region) corresponds to region with a combination of stress and environment, where minor species in a liquid or gas control the crack tip reaction. Segment III is independent from the environment: microstructure, material thickness and stress have a major influence on the crack growth rate within this segment.

If the ceramic is not ideally elastic, *i.e*., if it is toughened as happens in most composite ceramic systems, it generally exhibits *R-*curve behavior, meaning that the toughness rises within the crack length *a*, as shown in Figure 10b. Above a critical crack size (*a_c_*), steady state toughness is reached.

*R*-curve behavior is typical in ceramics toughened by second-phases (such as ZrO_2_, whiskers or fibers). The slope of the *R*-curve is an indication of the toughening rate during crack opening.

Increasing the toughness of ceramics is of critical importance, in particular if we consider their industrial manufacturing. Increased fracture toughness levels can significantly reduce the number of parts rejected during production and increase reliability during service, with a positive impact on the production costs. For this reason, there has been a considerable amount of research aimed at increasing the toughness of ceramics [73].

In this contest, nanocomposite ceramics are attracting increasing interest, since the dispersion of second-phase nano-grains can enhance the toughness of ceramics.

Crack bowing, which consists on the bowing of cracks between obstacles (*i.e.*, second phases) [73], is recognized as a toughening mechanism in composite ceramics: Lange [80] suggested that the interaction of a crack front with two or more resistant second-phase components can increase the fracture length and therefore the fracture energy along with the strength. Green [81] related the change in fracture energy to the fracture toughness change when crack bowing occurs by the following equation:

σ*_c_*/σ*_m_* = *K_c_*/*K_m_* = (*E_c_*Γ*_c_*/*E_m_*Γ*_m_*)^1/2^ (18)

where σ*_c_* is the stress needed to propagate a crack through a series of second phases, σ*_m_* is the matrix fracture strength, *K_c_* and *K_m_* are critical stress intensity factor, *E_c_* and *E_m_* are elastic moduli and Γ*_c_* and Γ*_m_* are fracture surface energy. Subscripts *c* and *m* denote composite and matrix, respectively.

Faber and Evans [82] further developed the crack bowing theory, by investigating the role of second phases having different morphologies, such as spheres, discs and rods, showing that the highest toughening is achieved in high aspect ratio disks within a brittle matrix. Faber and Evans [82] also investigated crack deflection phenomena, where crack deflection is different from crack bowing since the former produces a nonplanar crack, the latter a nonlinear crack front. Crack bowing and crack deflection have been recognized as the main toughening mechanisms where metal carbide second-phase are added to ceramic matrix, as shown in [82,83,84].

The change in fracture mode, from mixed inter/intragranular in monolithic ceramics to pure transgranular in nanocomposites also provides a toughening effect: the higher the extent of transgranular fracture, the higher the toughening [85]. Even more effective is the case of wavy fracture surface, occurring when a crack path is perturbed by subsequent particles embedded in a matrix. The wavy crack path enlarges the area of the crack surface and thus improves the toughness [85].

Recently, Awaji and co-workers [86] have underlined a relationship between the fracture toughness with the critical Frontal Process Zone (ZPS) size in nanocomposite ceramics. This model mainly applies to intra-type composites, in which the FPZ expansion ahead of a crack tip, founded on the Evans–Feber nanocrack mechanism [82], is considered the major factor responsible for the toughening increment in nanocomposites. Based on numerical modeling and experimental observations of Al_2_O_3_/SiC nanocomposites, dislocations are created in a temperature range from 600 to 1400 °C. During cooling, the thermal mismatch between matrix and second-phase particles allows the creation of sessile dislocations, which, at room temperature, will be localized close to the nanoparticles. Further annealing induces a dislocation rearrangement, leading to the development of dislocation networks or sub-grain boundaries around the particles [86]. When a main crack tip approaches this area, the dislocations act as nanocracks nuclei: the formation of many nanocracks expands the size of the FPZ of ceramics and hence improves the fracture toughness of the material. In addition, the sessile dislocations around the particles partially release the tensile residual stress in the grains and grain boundaries, thus strengthening the nanocomposite.

**Figure 11:**
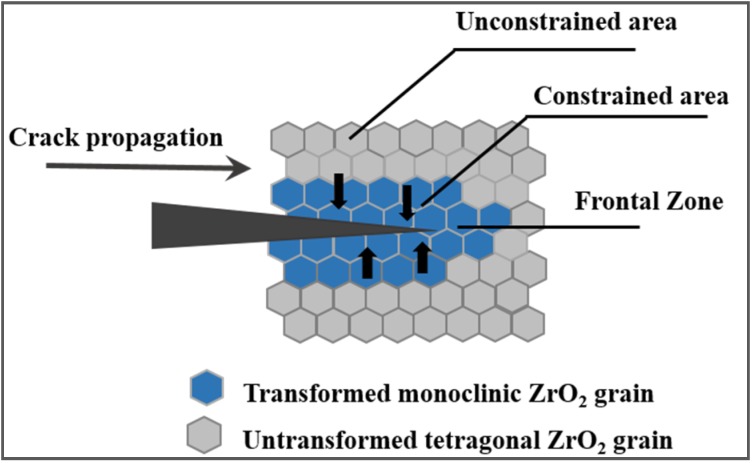
Schematic illustration of stress-induced phase transformation toughening.

Beside the toughening mechanisms previously described, transformation toughening deserves to be discussed [3,73]. Such behavior is presented by ceramics containing fine metastable ZrO_2_ grains, whose phase transformation is able to increase the crack propagation resistance. To explain such phenomenon, we should introduce the three polymorphisms of ZrO_2_, being progressively monoclinic (*m*), tetragonal (*t*) and cubic (*c*) at increasing temperatures. By adding proper stabilizers, such as yttrium oxide, the metastable *t* phase can be retained at room temperature, being able to re-transform into the stable *m* phase under an applied stress. As this martensitic transformation involves a volume expansion of ~3%–5% and a large shear strain (~7%) [73], it can induce a compressive stress at the crack tip and avoid the crack propagation, with a consequent toughening effect. A scheme of the transformation toughening mechanism is given in Figure 11.

Several conditions are required for transformation toughening to occur and the following parameters must be optimized:

(i) The ZrO_2_ particle size;
(ii) The stabilizing phase concentration;
(iii) The particle size distribution;
(iv) The particle-matrix thermal expansion mismatch.

In particular, it should be mentioned that unstabilized ZrO_2_ particles that are larger than a critical size will transform spontaneously during cooling, whereas particles which are too small do not transform, even under stress [73]. This highlights the key role of tailoring the microstructure during processing and sintering, in order to fully exploit the stress-induced phase transformation and maximize the mechanical properties.

Microcracking is a further toughening mechanism, derived from studies on ZrO_2_-toughened ceramics [87]. In fact, the relation between toughness increment in brittle solids and thermally or stress-induced microcracks was first demonstrated in Al_2_O_3_/ZrO_2_ system. Ceramic structures that contain localized residual stresses are susceptible to microcracking. Stresses can arise from phase transformation, thermal expansion anisotropy in monolithic ceramics and thermal expansion or elastic mismatch in composite ceramics. For microcracking to occur, stress-induced microcracking must be generated within a limited zone of stress concentration, near the crack tip. In addition, microcracking is possible only if the size of the dispersion exceeds a critical particle size [63].

Toughening is described by the Evans and Faber relations; in particular, for non-interacting second-phase spheres, under a residual stress (σ*_R_*), toughening is given by:

Δ*K_IC_* = *1.12V*σ*_R_h*^1/2^(1 + *ν*) (19)

where *V* is the volume fraction of spheres, *h* is the microcracking process zone and *ν* is the Poisson coefficient. In case of phase transformation associated to volume increase, as happens in martensitic transformation of zirconia, microcracks are expected to form radially from second-phase particles into the matrix. In this case, toughness increment is given by:

(20) $$\Delta K_{IC}=\frac{0.21Ee^{T}h^{\frac{1}{2}}(V+0.6V^{\frac{1}{3}})}{\left(1-\text{ν}\right)}$$

where *e^T^* is the volumetric strain of transformation, *E* the elastic modulus.

Transformation shielding has been recognized as the most effective toughening mechanism. In spite of this, experimental results show that when transformation is simultaneously accompanied by microcracking, the toughening effect is even more effective [88,89].

Finally, even if this review is mostly focused on particulate-reinforced ceramics, we should at least mention the important toughening effects of elongated second-phase or whiskers: here, toughening is mainly imputed to crack deflection, whisker pull-out and crack bridging mechanisms, as reviewed by Bengisu [73].

#### 3.2.3. High-Temperature Mechanical Properties

The presence of discrete, hard second-phase particles added to a ceramic matrix modifies the creep response of the materials, generally increasing its creep resistance. However, the scale of the effect depends on the volume fraction and morphology of the second-phase and how it is distributed in the matrix [90]. For instance, if the volume fraction of the second-phase is small, then the particles behave independently and the effect is only due to the disturbance of the flow field around the particles, resulting, for example, from a local increase in the effective path length for diffusion. As the particle fraction increases, antiparticle interactions begin to have effect. For example, local cluster of particles behave similarly to larger, single particles [90].

For two-phase composites, there are several equations which may express or predict their high temperature creep behavior: among them, we can recall the Raj and Ashby model [91]:

(21) $$\dot{\text{ε}}=C\frac{{\text{σ}}^{n}}{Vd^{p}r^{q}}\text{exp}(-\frac{Q}{RT})$$

where *V* is the second phase volume content, *r* is second phase grain radius, *q* and *n* are phenomenological exponents and *C* is a constant. This model applies to composites in which the second-phase can be considered rigid, and assumes that the hard second-phase particles limit the grain boundary sliding of the matrix. From the expression, we can see that the higher the second-phase volume fraction and the larger their size, the lower the creep rate.

Generally, for two-phase composites, especially for those with duplex microstructures, authors are not only interested in the dominant mechanism of deformation, but also try to find out which phase controls the creep behavior. Many investigations relate to Al_2_O_3_-based composites, in which SiC, YAG, ZrO_2_ and TiO_2_ are the mostly used second-phases. Such works demonstrate that the deformation behaviors are critically dependent on the physical and chemical properties of reinforcing particles or whiskers (including their content, morphologies and distributions), on the microstructures of composites (including grain sizes and shapes, pores, grain boundaries, interfaces), on the stress and temperature. During deformation, microstructural changes often occur, such as grain growth, changes of grain shape (grain elongation), texture development, formation of intermediate or intergranular phases, dislocation activity, vacancy nucleation and evolution, cavitation and evolution, formation and development of microcracks, *etc.* The study of such microstructural changes accompanying creep deformation is an essential aspect for analyzing creep deformation behaviors and creep mechanisms of composites [91].

Al_2_O_3_/SiC and Al_2_O_3_/YAG materials provided experimental evidence of the increased creep resistance of the nanocomposites as respect to monolithic Al_2_O_3_ [92,93,94,95]. The addition of SiC particles increases the creep resistance by one to two orders of magnitude in comparison to the monolithic Al_2_O_3_ [92,93,94,95]. The effect has been attributed to residual thermal stresses, created around SiC inclusions during cooling from the sintering temperature, as already reported. In particular, compressive residual stresses are created at the Al_2_O_3_/SiC interfaces (see Figure 9*b*), giving rise to stronger particle/matrix bonding as respect to matrix/matrix (*i.e.*, Al_2_O_3_/ Al_2_O_3_) bonding [96]. Furthermore, intergranular SiC particles exert a *pinning* effect on the Al_2_O_3_ grain boundary, inhibiting the grain boundary sliding and thus increasing the creep resistance [97]. YAG is considered one of most creep resistant oxide ceramic [98] and its addition to Al_2_O_3_ provides a decrease of the creep rate of one order of magnitude [94]. A previous study [99] showed that the creep rate was controlled by YAG (the more creep-resistant phase) and suggested a diffusional Nabarro–Herring mechanism.

## 4. Synthesis of Nanocomposite Ceramic Powders

As previously reported, the addition of nanosized or ultra-fine second-phases can induce significant increments in the materials mechanical properties, in terms of hardness, strength, fracture toughness and creep resistance.

However, a key point to achieve excellent mechanical properties in nanocomposite ceramics is a proper design and fabrication process to yield tailored sintered micro/nanostructures. When designing a composite structure, besides considering the chemical composition of matrix and second-phase, the amount, distribution, morphology of the reinforcement phase should be properly evaluated. A key point is achieving a homogenous distribution of the second-phase in the matrix material, but this is still challenging when nanocrystalline particles are used, due to their extremely high specific surface and, hence, intrinsically high tendency to agglomeration. This means that all manufacturing steps towards the elaboration of nanocomposite ceramics should be carried out with particular care, from the synthesis of the composite powders, to the forming of green bodies and their sintering. Although the processing and sintering routes of nanoceramics have been revised in some previous papers [1,100], the role of synthesis on the microstructure and properties of sintered composite ceramics have been poorly discussed in literature.

The most investigated structural ceramic nanocomposites are Al_2_O_3_/SiC and Si_3_N_4_/SiC. However, many other different phases such as TiN, TiC, TiO_2_, ZrO_2_, Cr_3_C_2_, YAG, can be used as nanoreinforcements in Al_2_O_3_, Si_3_N_4_, MgO, mullite or SiAlON ceramic matrix. In the following, the mainly used synthesis routes for the above composite systems are reviewed. First, the traditional mixing and milling method of single-phase ceramic powders to produce a composite feedstock, is described. Then, the more advanced synthesis methods used for directly producing composite powders are described. For sake of clarity, Table 1 summarizes direct composite powder synthesis methods and provides some examples of compositions already developed.

**Table 1:** Synthesis methods of nanocomposite ceramic powers and examples of developed compositions.

Synthesis Route	Type of Composite	Composition	References
Mechanochemical	Oxide/oxide	HA/MgTiO_3_/MgO; β-CP/MgTiO_3_/MgO	[101]
	Oxide/non-oxide	Al_2_O_3_/ZrB_2_/ZrO_2_; Al_2_O_3_/TiB_2_	[102,103]
	Non-oxide/non-oxide	B_4_C/SiC, NbC/NbB_2_	[104,105]
Polymer precursor	Oxide/non-oxide	Al_2_O_3_/SiC; Mullite/SiC	[71,106,107,108]
	Non-oxide/non-oxide	ZrC/SiC; Si_3_N_4_/SiC	[109,110,111,112,113,114,115,116]
Vapor Phase	Oxide/oxide	ZrO_2_/SiO_2_; TiO_2_/V_2_O_5_	[71,117]
	Non-oxide/non-oxide	Si_3_N_4_/SiC	[71]
SHS	Oxide/non-oxide Non-oxide/non-oxide	Al_2_O_3_/SiC; Mullite/TiB_2_	[118,119] [120,121,122,123,124]
		Si_3_N_4_/TiN;Si_3_N_4_/MoSi_2_; Si_3_N_4_/SiC;	
		TiN–SiC–Si_3_N_4_; ZrB_2_–SiC–ZrC–ZrSi	
Sol-gel	Oxide/oxide	Al_2_O_3_/ZrO_2_;Al_2_O_3_/Y_3_Al_5_O_12_;	[125,126,127,128,129,130]
		Mullite/ZrO_2_; Mullite/TiO_2_	
	Oxide/non-oxide	Al_2_O_3_/SiC; Mullite/SiCAlN/BN	[131,132,133,134]
	Non-oxide/non-oxide	Mullite/SiCAlN/BN	[135]
Co-precipitation	Oxide/oxide	Al_2_O_3_/ZrO_2_;Al_2_O_3_/Y_3_Al_5_O_12_; ZrO_2_/Gd_2_O_3_; Al_2_O_3_/LaAl_11_O_18_; Ca_10_(PO_4_)_6_(OH)_2_]/Fe_2_O_3_/Mullite/Al_2_O_3_.	[34,136,137,138,139,140,141,142,143]
Solution combustion/	Oxide/oxide	Al_2_O_3_/ZrO_2_; CeO_2_–M_x_O_y_; MO_x_–ZnO;	[73,144,145,146,147,148,149,150,151]
Spray decomposition		γ-Fe_2_O_3_–TiO_2_; Al_2_O_3_/ZrO_2_/MgAl_2_O_4_	
Surface modification route	Oxide/oxide	Al_2_O_3_/ZrO_2_; Al_2_O_3_/Y_3_Al_5_O_12_; Al_2_O_3_/Mullite; Al_2_O_3_/SiO_2_; ZrO_2_/MgAl_2_O_4_; Al_2_O_3_/ZrO_2_/Y_3_Al_5_O_12_; ZrO_2_/Al_2_O_3_/SrAl_12_O_19_	[14,16,21,62,70,88,152,153,154,155,156,157,158,159]
	Oxide/non-oxide	SiC/Al_2_O_3_; SiC/Y_2_O_3_	[160]

### 4.1. Conventional Powder Route

Most ceramic composite powders are currently produced by mechanical mixing of the constituent phases. This route involves the selection of raw materials, paying particular attention to the primary particle size, size distribution, agglomeration degree and purity. High purity powders are necessary to avoid the formation of a secondary phase during sintering. A wide particle size distribution on one hand leads to a higher packing density in the green bodies. On the other hand, the control of the microstructural development during sintering could be difficult because the larger grains can coarsen, including the smaller ones. At the same time, the particle size influences the final grains size and the densification rate (see Section 2.1 and Section 3.1): due to the higher specific surface, the densification rate increases as the particle size decreases. In addition, if the powder is characterized by a certain agglomeration degree, the packing in the green density will be heterogeneous, giving rise to differential sintering rates and to heterogeneous microstructures. In addition, a not homogeneous particle packing can produce large flaws, with a negative effect on the strength of the sintered composite, as shown in Section 3.2.1. For this reason, ultra-fine, loosely dispersed powders are needed for both matrix and reinforcing phases, to guarantee effective mixing and satisfactory dispersion of second-phase grains in the final product. In addition, the crystalline phase of the raw powder can play a role on the densification behavior and microstructural development. In the case of Al_2_O_3_-based composites, both transition Al_2_O_3_ (such as γ-Al_2_O_3_) and α-Al_2_O_3_ can be used. The main advantages in the use of γ-Al_2_O_3_ powder are its finer particle size, the loosely packed morphology and high surface area. However, the transformation from metastable, transition phases (*i.e.*, γ and θ phase) to α-Al_2_O_3_ occurs by a nucleation and growth mechanism. This transformation is accompanied by a volume reduction of about 10.2%, due to the higher density of the α-phase and a change in grain morphology. In fact, a well-known vermicular morphology is produced, due to the high temperature finger growth of α-grains into the θ-Al_2_O_3_ matrix, entrapping a network of large, elongated pores. Therefore, the final stages of sintering require very high temperature to achieve full densification, thus leading to a significant grain growth [161]. For this reason, α-Al_2_O_3_ particles are usually the preferred choice as raw materials for both monolithic Al_2_O_3_ parts and related composite materials.

The homogenization of the powder mixture can be achieved by either wet ball or attrition milling, carried out in aqueous or organic media. Al_2_O_3_/SiC composites are frequently prepared by this conventional powder mixing route, by using ball-milling as well as ultrasonic dispersion [71]. The most critical step is drying, because of the risk of agglomerate formation. An infrared heat lamp or freeze drying was successfully used to avoid such a drawback [71].

In spite of such expedients, the major drawback of this method is the inability to achieve an optimal distribution of ultrafine (~50 nm) second-phase grains in a matrix powder, due to agglomeration and dispersion problems. In addition, the milling step can induce a certain pollution of the powders, derived from the milling media. For these reasons, new synthesis methods to directly produce nanocomposite powders have been developed, as described in the following.

### 4.2. Mechanochemical Synthesis

This synthesis implies high-energy milling techniques and is frequently carried out under controlled atmospheres. It has been used for complex compositions and for Al_2_O_3_ reinforced by intermetallic compounds [162]. Composite powders are prepared as well, as shown in [103,104,105] for oxide, non-oxide and mixed oxide/non-oxide materials. Fracture mechanics theory suggests that the smallest possible fragments that can be created by repeated, high-energy milling of particles are in the 5–100 nm range. Indeed, 100 nm particles are rarely (if ever) observed in ceramic milling tests. What commonly happens is the creation of larger particles which themselves contain ultrafine grains [1]. In addition, *in situ* localized phase transformations have been also observed, in response to localized damage.

The major drawbacks associated with this synthesis method are the inability to produce discrete nanoparticles in the finest size range and the tendency to contaminate powders with the milling media used to grind them, particularly when extensive and repeated milling cycles are carried out.

### 4.3. Polymer Precursor Route

Polymer-derived ceramics is an alternative to conventional powder-based ceramics. Here, a pre-ceramic, mostly a Si-based polymer (e.g., polysiloxane, polysilazane, or polycarbosilane), is used as starting material. The cross-linked polymer can be transformed into a ceramic material by a controlled thermal decomposition treatment (pyrolysis).

This method has been applied to the elaboration of various composite systems, as shown in Table 1. In the case of Al_2_O_3_/SiC composites, the Si-containing polymeric precursor (polycarbosilane) is coated onto a surface-modified Al_2_O_3_ powder and pyrolysed at 1500 °C to produce ultrafine SiC particles (size less than 20 nm) [106]. The powder is hot-pressed at 1700 °C, forming a fully dense nanocomposite. By comparing the sintered microstructure of Al_2_O_3_/SiC prepared by conventional powder processing and polymer precursor route, it was shown that this latter technology provides much finer, uniformly distributed, SiC nanopowders [71].

The polymer precursor route has also been employed to synthesize Si_3_N_4_/SiC nanocomposite powders. Two methods have been tested. In the former, mixtures of Si_3_N_4_ powder, sintering aids and the polymer (a polymethylphenylsilane) are attritor milled, cold isostatically-pressed, pyrolysed at 1000 °C under Ar and, finally, pressureless sintered at 1850 °C in N_2_. The resulting Si_3_N_4_/10_wt_% SiC nanocomposite (fired density; 96.7%) consisted of micron-sized Si_3_N_4_ grains with well-dispersed SiC particles [110]. In the latter, polymethylsilazane is converted through crosslinking and pyrolysis at 1000 °C to an amorphous Si-C-N powder [110,111]. Pressureless sintering of the cold isostatic-pressed powder mixture at 1750 °C led to a composite of 97% density and a SiC content of 24_wt._%. The microstructure of the final composite was nanocrystalline for both Si_3_N_4_ and SiC phases with *d*_50_ = 0.2 μm, showing a homogeneous SiC distribution [111,115,116].

**Figure 12:**
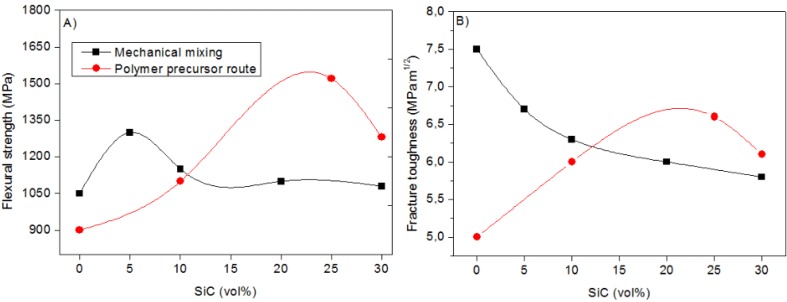
(**a**) Flexural strength and (**b**) fracture toughness of S_3_N_4_/SiC composite as a function of the SiC volume content. Black symbol: powder by conventional mixing and milling method [113]; red symbol: powder by polymer precursor route [114]. Redrafted from [71].

Sternitzke’s review [71] provides a comparison between the mechanical properties of Si_3_N_4_/SiC nanocomposite prepared by conventional powder route [113] and by the polymer precursor method [116] (Figure 12). In particular, in the former case, we can see that the strength of Al_2_O_3_ significantly increases by adding 5_vol._% of SiC, but it decreases again for further additions. The toughness showed a continuous decrease by increasing the SiC content. This was imputed to a decrease of the sintered density and to agglomeration of SiC particles, giving rise to a poor distribution of the second-phase in the composite. Better results were obtained by the polymer precursor method, showing a continuous increase of both properties for high SiC contents (up to 20_vol._%), due to a more homogeneous distribution of SiC particles in the nitride matrix.

### 4.4. Vapor Phase Reaction Technique

Vapor phase methods play an important role in the commercial production of commercial powders, including both oxide and non-oxide powders. This category is dominated by gas phase condensation technique, in which a solid is evaporated by Joule heating, to form a supersaturated vapor, out of which small scale particles subsequently condense. The supersaturated vapor can be also achieved by other routes (electron beam evaporation, laser ablation, magnetic sputtering, arc discharge, *etc.*) leading to a variety of particles size, shape and compositions. The physical processes involved are chemical reaction, mass transfer, nucleation, coagulation and condensation. The method allows the production of nanocrystalline powders, eliminating the need of a subsequent calcination step, characterized by high purity and controlled particle size distribution. The nature of the gas determines the type of powder produced: inert gas, like helium, is usually used to synthesize non-oxide powders; otherwise, oxygen-containing gases can be used to produce high purity metal oxide powders. In this case, a subsequent annealing process at high temperatures is often required to complete the oxidation. NH_3_ (g) is usually used to prepare metal nitrides, whereas alkanes or alkenes are used as a source of carbon to prepare metal carbides [24].

Si_3_N_4_/SiC nanocomposite powders have been prepared by vapor phase pyrolytic reaction [71]. [Si(CH_3_)_3_]_2_NH or [Si(CH_3_)_2_NH]_3_, are mixed with NH_3_, under N_2_ used as carrier gas, and then passed into a reaction chamber at 1000 °C. The amorphous powder is collected and crystallized to Si_3_N_4_/SiC at 1500 °C for 6 h. The carbon content in the as-received powder and thus the SiC content in the crystallized powder can be adjusted by the NH_3_ content with a maximum value of approximately 34_vol_.% SiC in the final nanocomposite. The resulting submicron powder is highly reactive and can react with oxygen or water to generate heat or even ignite. Therefore, an immediate heat treatment at 1350 °C for 4 h, under Ar, is necessary [71].

ZrO_2_/SiO_2_ powder has been produced by vapor decomposition route, in which the precursors (namely, [Si(OC_2_H_5_)_4_] and zirconium *tert*-butoxide) are introduced in the reactor as vapors, obtained by bubbling a carrier gas through the precursor solution. The resulting vapor-saturated carrier gas is introduced into a plasma, where thermal decomposition takes place. Outside the plasma region, nucleation and growth of the ceramic powders occur. In the final product, small ZrO_2_ grains were trapped in SiO_2_ powders [73].

### 4.5. Self-Propagating High-Temperature Synthesis (SHS) and Combustion Synthesis (CS)

SHS involves the ignition of powder mixtures that exhibit an exothermic reaction and produce temperatures in the range 1000–3000 °C under adiabatic conditions. Besides powders production, this technique is also used for direct production of ceramic bodies. In addition, by this route, both single and multiphasic ceramic compositions can be produced. In fact, the method has been applied for *in situ* synthesizing of a number of refractory materials and composites, as reported in Table 1. An example is provided by the following reaction, used to prepare cermet compositions:

*w*Ti (s) + *x*B (s) + *y*C (s) + *z*Cu (s) → *l*TiB_2_ (s) + *m*TiC (s) + *z*Cu (s) (22)

TiN/Si_3_N_4_ composites have been combustion synthesized using a mixture of elemental Si and Ti in a nitrogen atmosphere [123]. High exothermicity of the nitridation process is frequently accompanied by melting of components in the interaction zone, which impedes nitrogen infiltration from an external medium to the combustion front, resulting in incompleteness of the reaction. To prevent this undesirable phenomenon, an inert diluent (mainly TiN and/or Si_3_N_4_) is usually introduced into the initial mixture. Another approach implies the use of initial compositions, containing compounds instead of individual elements. For example, TiN–SiC–Si_3_N_4_ composites were synthesized from a TiSi_2_–SiC mixture via a combustion reaction under a nitrogen pressure of 130 MPa [122]. Different titanium silicides (TiSi, Ti_5_Si_3_, TiSi_2_) have been tested as initial raw materials [120]. Thermodynamic analysis and relevant experiments have shown that at relatively low nitrogen pressure (up to 5 MPa) Ti_5_Si_3_ is the most suitable compound for this purpose.

In a recent work [124], bi-, tri- and tetra-phasic powders, such as ZrC/SiC, ZrB_2_/SiC/ZrC, ZrB_2_/SiC/ZrC/ZrSi, were synthesized as fine powder *via* combustion synthesis using a mixture made by ZrSiO_4_, Mg, C, B and NaCl as raw materials. In particular, Mg was used to reduce ZrSiO_4_, whereas NaCl was used as a diluent to control the particle size and phase composition of the composite powders. The authors showed that the combustion temperature and particle size decreased by increasing the NaCl amount in the starting mixture.

### 4.6. Solution Techniques

#### 4.6.1. Sol-Gel

Sol-gel processing is the most popular kind of liquid-phase processing of nanocomposite powders.

Inorganic salts or metal-organic compounds are used for the sol preparation. Then, by hydrolysis and condensation reactions, the sol is converted into a gel (at a point called sol-gel transition) that has to be dried, to eliminate the exceeding liquid phase. When dried, the gel shrinks and transforms to the desired phase. This route allows obtaining complex shapes, directly from the gel state, controlling the homogeneity of chemical composition and lowering the processing temperatures. In addition, by controlling the gelation parameters and subsequent thermal treatments, it is possible to tailor the microstructure.

Although this process is quite viable, raw materials, especially the organometallics, can be expensive and often sensitive to moisture. Moreover, during the synthesis of multi-cation materials, the hydrolysis and condensation reactions have to be carefully controlled, in order to avoid segregation phenomena: suitable solution pH, temperature and reactant concentration have to be set up.

The products may also be amorphous, requiring high-temperature calcination where agglomerates may coarsen the microstructure.

An example of Al_2_O_3_/ZrO_2_ composite powder developed through sol-gel process was reported by Jayaseelan [125]. First, stable (hydrous) boehmite and oxalate sols were prepared. In particular, zirconyl oxalate, cerium zirconyl oxalate and yttrium zirconyl oxalate were prepared, with the aim of producing un-stabilized, ceria-stabilized or yttria-stabilized ZrO_2_/Al_2_O_3_ composites, respectively. The sols were then mixed in proper ratio to obtain the precursor of the desired composition. The sol was then converted into a gel by stabilization, and the gel dried. Then, the amorphous precursors were calcined at different temperatures in order to investigate the effect of the calcination treatment on phase composition and density. After calcination and pressureless sintering, a homogeneous microstructure was obtained, in which nanometric/submicrometric zirconia grains were located at the junctions of the Al_2_O_3_ grains and grain boundaries.

A recent study of Naga [126] compares the microstructural development and mechanical properties of (Y,Ce)-ZrO_2_/Al_2_O_3_ composites prepared by sol-gel and mechanical mixing methods. The results indicate that, due to the higher homogeneity achieved from the sol-gel technique, the sol-gel derived composite showed higher bulk density, lower apparent porosity and a finer microstructure with respect to the mechanically-mixed composite. This had an important effect on the mechanical properties, as shown in Table 2.

**Table 2:** Physical and mechanical properties of sol-gel and mechanical mixing-derived composites, sintered at 1650 °C for 1 h [126].

Synthesis Route	Bulk Density (g/cm^3^)	Vickers Hardness (H_v_)	Bending Strength (MPa)
Sol-gel	3.90	1385.5	219.8
Mechanical mixing	3.62	1076.5	184.6

The sol-gel process can be also used to synthesize non-oxide-based nanocomposite powders, as shown in [131,132,133,134,135]. In the case of Al_2_O_3_/SiC nanopowders, the SiC nanopowder is dispersed in a suitable liquid medium to create a stable suspension and mixed with an Al_2_O_3_ precursor, such as an AlCl_3_.6H_2_0 solution [132,133]. After gelation and drying, the xerogel containing SiC nanoparticles is calcined, crushed, sieved and finally used for preparation of nanocomposites.

Strutt *et al.* [135] synthesized an AlN/BN composite powder. The method involves the low temperature conversion of an aqueous solution of aluminum, boron, and nitrogen containing compounds to form intermediate precursor material. This material is essentially a gel, and subsequent high temperature conversion produces AlN/BN composite. The material resulted to be nanoporous, containing a distribution on AlN and BN nanoparticles.

#### 4.6.2. Co-Precipitation

In the precipitation technique, the solubility of the desired cations dissolved in an aqueous solution is exceeded by evaporation of the liquid or by adding a chemical precipitant agent. Thus, the precipitation of metal hydroxides is promoted. On one hand, the modification of pH and temperature of the solution allows controlling nucleation and growth mechanisms and consequently the particles morphology. On the other hand, a common problem for co-precipitation is the achievement of a suitable condition for the simultaneous precipitation of all the species present in solution.

Precipitation is a nucleation and growth process with Arrhenius control of the kinetics. High nucleation and slow growth rates are usually required to keep the particles powders small.

Rana *et al*. [136] synthesized Al_2_O_3_/ZrO_2_ composite powders starting from zirconium and aluminum chlorides. Three different processing routes, precisely gel precipitation (GPT), precipitation (PPT) and washed precipitation (WPT) were investigated. In all three cases, aqueous ammonia was added to induce precipitation. During synthesis, the pH was maintained in the range 6–6.5 (gelation point) for GPT, whereas for the other two routes precipitation was carried out at a higher pH, in the range 8.7–9.1. The difference between the PPT and WPT routes lays in the washing process: in the former case, the precipitate was separated by the liquid and then dried; in the latter, it was washed with hot water and alcohol before drying. These different processing routes affected the crystallization temperature of the amorphous powder as well as the phase evolution of Al_2_O_3_ and ZrO_2_ phases during calcination. In fact, while GPT and PPT powders crystallized at 350 °C, no crystallization was observed in WPT powder till 650 °C, this latter being the only product able to produce pure tetragonal zirconia phase. The agglomerate size was largest for GPT powder, smallest for WPT. The former produced hard agglomerated powder, while WPT produced soft agglomerates with low agglomeration strength, giving rise to compacts with good green density and able to sinter to a high density at lower sintering temperature. WPT sample sintered at 1550 °C reached the maximum Vickers hardness and bending strength values.

A co-precipitation route was also used by Balmer *et al.* [137] to develop ZrO_2_/Al_2_O_3_ nanocomposites having plate-like grains. The composite was prepared starting from an aqueous solution containing aluminum nitrate hydrate and zirconium acetate. Then, to avoid the selective crystallization of the aluminum nitrate during solvent removal, the solution was atomized onto a Teflon-coated aluminum substrate at 250 °C. In this way, an amorphous powder was prepared, which was then submitted to thermal treatments. The authors stated that it was possible to control the scale and development of the lamellar microstructure by controlling the composition and heat treatment conditions. At temperatures below 1100 °C the tetragonal zirconia and γ- Al_2_O_3_ phases with a grain size ranging between 40 and 100 nm were found. At 1200 °C large (2–4 µm) and thin plate-like Al_2_O_3_ grains, containing nanometer zirconia inclusions, were formed inside the zirconia matrix. According to the authors, the lamellar morphology is a consequence of the strain energy and diffusional phenomena associated with the γ → α phase transformation.

Han *et al*. [138] investigated the role of precipitant and drying method on Al_2_O_3_/ZrO_2_ powders synthesizes by co-precipitation. In particular, NH_4_HCO_3_, NH_4_OH, and (NH_3_)_2_CO_3_ were used as precipitant agents. Three drying methods were compared: vacuum drying, spray drying and freeze drying. The results showed the proper use of NH_4_HCO_3_ as precipitant and freeze drying as drying method, since the Al_2_O_3_/ZrO_2_ powders were characterized by a particle size distribution in the 30–60 nm range, with an average sphere diameters of 47 nm.

Wang *et al.* [139] prepared Al_2_O_3_/5_vol_.%YAG by comparing three different methods: (i) co-precipitation, (ii) precipitation of Al(OH)_3_ in a slurry containing YAG particles; (iii) traditional milling of Al_2_O_3_ and YAG powders. All materials reached full density after hot pressing in the range 1500–1650 °C. Both wet-chemical methods (i) and (ii) gave rise to a very homogeneous distribution of YAG grains in the matrix. Highest mechanical properties were determined for the co-precipitated powder, whereas lowest values were determined for the mixed material, as shown in Table 3.

**Table 3:** Mechanical properties of Al_2_O_3_/YAG powders prepared by (i) co-precipitation; (ii) precipitation of Al(OH)_3_ in a YAG slurry; (iii) traditional milling [139].

Synthesis Route	Sintering Temperature (1500 °C)		Sintering Temperature (1650 °C)	
	Bending Strength (MPa)	Fracture Toughness (MPa√m)	Bending Strength (MPa)	Fracture Toughness (MPa√m)
Co-precipitation	604 ± 25	5.0 ± 0.5	402 ± 21	4.1 ± 0.1
Precipitation	485 ± 28	4.2 ± 0.5	284 ± 4	4.0 ± 0.1
Milling	432 ± 140	4.2 ± 0.6	111 ± 14	3.5 ± 0.4

**Figure 13:**
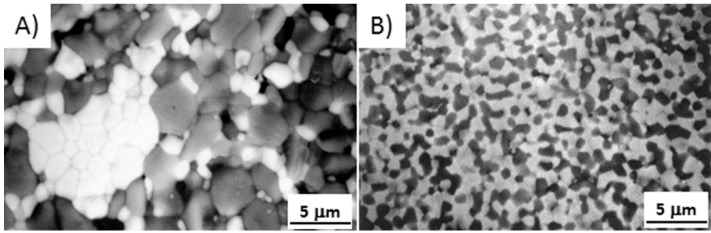
SEM micrographs of Al_2_O_3_/50_vol._%YAG sintered materials, prepared by (**a**) traditional mixing route and (**b**) co-precipitation, both sintered at 1600 °C for 3 h.

Finally, Figure 13 compares the microstructure of Al_2_O_3_/50_vol_.%YAG prepared by (a) powder mixing and (b) co-precipitation, both sintered at 1600 °C for 3 h, [34], showing the presence of cluster of YAG grains in the former composite, imputable to a not sufficient mixing of the powders during the milling step. On the opposite, an optimum distribution of both phases is achieved in the second material, which also shows a significantly finer microstructure.

#### 4.6.3. Spray Decomposition

Spray decomposition or pyrolysis is a versatile technique for producing ceramic materials with a wide variety of particle morphologies, sizes and compositions. A carrier gas flows through an aerosol generator (ultrasonic atomizer, spray gun, nebulizer, *etc.*) where an aerosol or spray of the desired precursor solution is produced and directed into the furnace. The synthesis steps involve: (i) the formation of the precursor aerosol; (ii) the evaporation of the solvent and solute precipitation; (iii) the reaction or thermal decomposition of the solute to yield the ceramic powder.

Aqueous solutions are usually used because of their low cost, safety, and the availability of a wide range of water-soluble salts. The advantages of this method include the production of high-purity nano-sized particles, with a high degree of homogeneity.

The disadvantages include the need for large amounts of solvents and the difficulty to scale-up the production. The use of large amounts of non-aqueous solvents increases the production expenses because of the high cost of pure solvents and the need for proper disposal.

Al_2_O_3_/ZrO_2_ powders have been produced by this method [73], presenting the advantage of precise control of powders stoichiometry and limited agglomeration. The disadvantage is poor control of powder morphology that usually results in porous or doughnut-shaped particles.

#### 4.6.4. Solution Combustion

This method is based on the gelling and subsequent combustion of an aqueous solution containing metal salts (usually nitrates) and some organic fuel. The combustion process is due to an exothermal redox reaction between nitrate ions and the fuel. The large volume of gases produced during the reaction promotes disintegration of the over-inflated precursor gel yielding a nanocrystalline powder after calcination.

Nanocrystalline Al_2_O_3_/ZrO_2_ powders were produced by this method, using metal-nitrate as the oxidizer and urea as the fuel. The average particle size of α-Al_2_O_3_ was 40 nm, while a smaller value was determined for the tetragonal-zirconia particles. The composite powders were densified by hot isostatic pressing at 1200 °C into a dense (99%) compact, with average grain size < 100 nm [145]. Tahmasebi *et al*. [146] observed that the use of microwave heating during the solution combustion process of Al_2_O_3_/ZrO_2_ powders allowed decreasing the particle size as compared to the conventional process. In the case of microwave-assisted synthesis, ultra-fine powders (<20 nm), with narrower size distribution and a high homogeneity of distribution of ZrO_2_ particles in Al_2_O_3_ matrix were successfully produced.

Al_2_O_3_/ZrO_2_/MgAl_2_O_4_ powders have been prepared by this method [150], showing that this route allows the production of fine and highly pure oxide powders. The method requires simple equipment, which allows saving time, energy and money. Aluminum and magnesium nitrates as well as zirconyl nitrate were used as raw materials. The salts were dissolved in water and mixed with urea; the combustion process was carried out on hot plate at 300 °C. The powder was finally calcined at 1200 °C. The method was successful in producing fine powders, with controlled tetragonal zirconia content and the required purity.

### 4.7. Surface Modification Methods

The previous paragraphs highlight that the traditional powder mixing is a simple procedure but often leads to inhomogeneity of the final microstructure. On the other hand, the chemical routes allow a better control of the microstructure but are more complex to manage.

For these reasons, innovative procedures consisting in the surface modification of a commercial powder with the second-phase precursors have been exploited in recent years. Surface modification routes can be considered as a compromise between the powder mixing technique and the chemical ones and allow an improved control of the final microstructure. In fact, the close mixing between the matrix ceramic (nano)particles and the metal ions, precursors of the second-phases, is realized at atomic/nano level, assuring an excellent distribution of the second-phases in the composite powders. On the opposite, in the milling methods, the second-phases are in the form of a solid precipitates or solid particles, leading to a less effective mixing with the matrix powder.

Cortesi and Bowen [152] developed Al_2_O_3_/ZrO_2_ composite powders by coating commercial α-Al_2_O_3_ powders with zirconium alkoxide, in *n*-propanol. In order to promote the zirconium alkoxide hydrolysis, a solution of water in *n*-propanol was prepared. Both the Al_2_O_3_/Zr-alkoxide suspension and the water/n-propanol mixture entered a continuous flow reactor, in which hydrolysis of the alkoxide took place, converting it into oxyhydroxide. A similar method was employed by Schehl *et al*. [153], in which organic precursors (metal-alkoxides) of the second-phases were grafted on the surface of a commercial Al_2_O_3_ powder. Through an organic media (typically ethanol), a substitution reaction between the metal alkoxide and the OH groups took place on the particles surface. The modified powder was then dried under magnetic stirring at 70 °C and thermally treated to obtain a composite powder [153]. This method was successfully applied to the preparation of Al_2_O_3_-based composites, containing YAG, ZrO_2_ and mullite as second phases, showing in all cases a fine and homogeneous microstructure, with well-distributed second-phase nanoparticles in the Al_2_O_3_ matrix. The mechanical properties of Al_2_O_3_/ZrO_2_ composites obtained by this colloidal route and by classical mechanical mixing method have been compared [153,155]. The finer microstructure proper of the composite processed by colloidal route leaded to a higher stress-intensity factor for crack growth as well as to higher stability of tetragonal zirconia grains against hydrothermal ageing.

Besides the method investigated by Schehl *et al.* [153], based on the use of organic precursors, also inorganic salts were used to coat the surface of commercial Al_2_O_3_ particles and to induce the crystallization of second-phases upon calcination. Yuan *et al.* [156], for instance, added cerium and aluminum nitrates to the isopropanol suspension of ZrO_2_ powder, in order to obtain ceria-stabilized ZrO_2_/2_wt._%Al_2_O_3_ composites. The as-obtained suspension was mixed for 48 h on a multidirectional mixer and then water and alcohol media were removed by means of a rotating evaporator at 95 °C, thus promoting the formation of the desired final phases. The dried powder was subsequently calcined in air at 800 °C for 1 h in order to obtain the Al_2_O_3_-doped CeO_2_-coated ZrO_2_ nanopowder.

**Figure 14:**
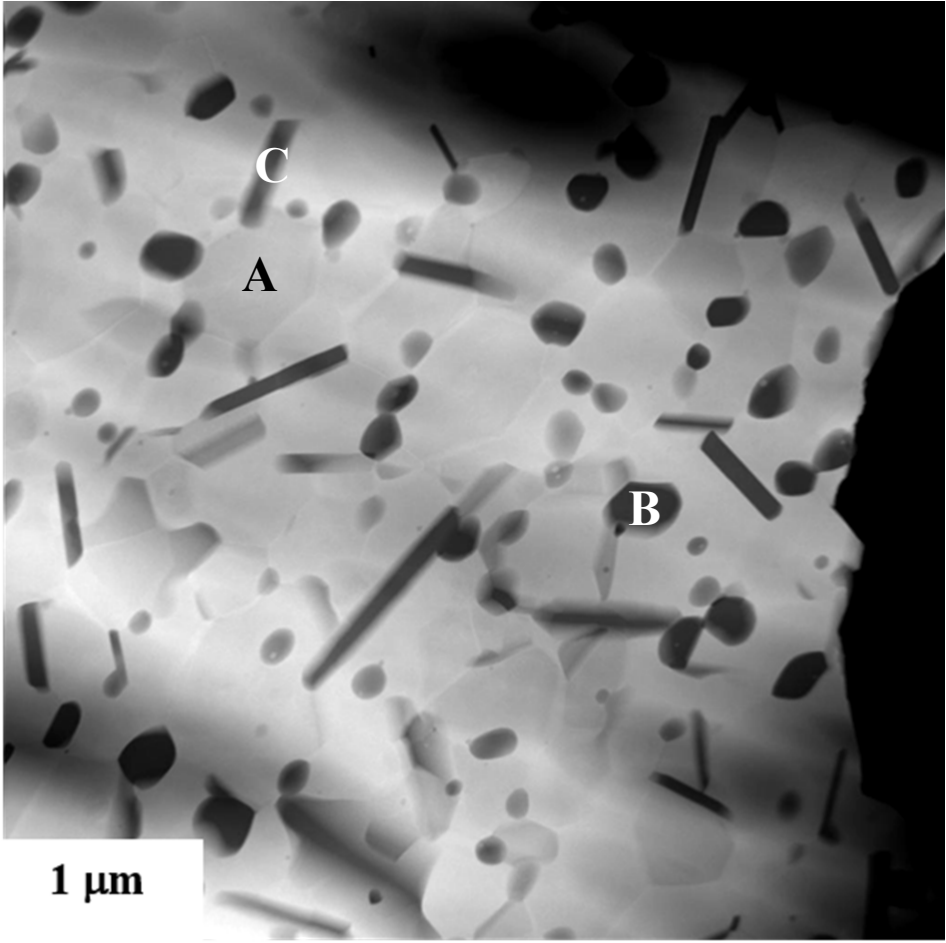
TEM micrograph of Zr_0.89_Ce_0.11_O_2_/Al_2_O_3_/SrAl_12_O_19_ composite, produced by the surface modification method. Letters A, B and C denote the Ceria-stabilized ZrO_2_, the α-Al_2_O_3_ and the SrAl_12_O_19_ phases, respectively, as determined by EDX analysis.

More recently, an alternative method in the domain of the “surface modification route” of commercial powders was developed [88,157]. This method shows some advances as respect to the previous techniques. In fact, here only inorganic precursors and aqueous media are used, making this strategy much simpler and potentially transferable to a pre-industrial scale production. A second difference lays in the mixture drying method, which is here performed by means of a “flash” drying, such as atomization, in which the liquid medium is converted into fine droplets and instantaneously evaporated. This step has a key role in the processing, since the homogeneity of the above mixture is “frozen” in the dried product, completely avoiding the segregation of the metallic dopants, as can occur by slow drying in an oven. The method was successfully applied to Al_2_O_3_-based bi- and tri-phasic composites [21] and, more recently, successfully exploited for the elaboration of ZrO_2_-based composites with complex compositions and microstructures. In particular, the method was applied to the elaboration of Zr_1__−*x*_Ce*_x_*O_2_/8_vol._%Al_2_O_3_/8_vol._%SrAl_12_O_19_ (with *x* ranging between 10 and 11.5), in which α-Al_2_O_3_ and SrAl_12_O_19_ grains were characterized by rounded and elongated morphology, respectively [14]. Precursors for the desired second-phases were Al(NO_3_)_3_ 9H_2_O and Sr(NO_3_)_2_. In order to obtain different ceria contents in the zirconia lattice, ammonium cerium (IV) nitrate, (NH_4_)_2_[Ce(NO_3_)_6_] was employed as ceria precursor and added as well. The nitrates were dissolved in distilled water and then drop-wise added to the dispersed zirconia suspension. After mixing for 2 h, the suspension was spray dried. The powder was then pre-treated at 600 °C, for 1 h, in order to decompose the synthesis by products, and then thermally treated at 1150 °C for 30 min, in order to approach the crystallization temperature of the second-phases. Green bodies produced by slip casting were sintered at 1450 °C/1 h, yielding full densification. The method successfully allowed obtaining the desired, complex microstructural and compositional features in the sintered composite [14], as shown by TEM/EDX analyses. In fact, as we can see in Figure 14, an optimal distribution of both second-phases in the zirconia matrix was achieved. Moreover, all second-phases had the expected composition and morphology, and the correct amount of ceria was tuned inside the zirconia lattice.

### 4.8. Industrial Production of Ceramic Composite Powders

All examples provided in the previous paragraphs refer to laboratory-scale powder production. In fact, the main industrial suppliers of ceramic powders still limit their production to mono-phase oxide or non-oxide powders. Possibly, complex compositions as well as bi- or multi-phasic compositions are supplied on demand. Few exceptions are represented by the Tosoh Corporation (Tokyo, Japan), which supplies an ATZ grade powder, consisting on a mixture of 80_wt._% ZrO_2_ (stabilized by 3_mol._%Y_2_O_3_)/20_wt._%Al_2_O_3_, produced by hydrolysis process [163]. Goodfellow Cambridge Ltd. (Huntingdon, England) also offers both Al_2_O_3_-toughened ZrO_2_ and ZrO_2_-toughened Al_2_O_3_ composite powders [164]. Phelly Materials Inc. (Upper Saddle River, New Jersey, USA) provides different mixtures of oxide powders in forms of granules, suitable for coating applications, like TiO_2_/Nb_2_O_5_, Al_2_O_3_/ZrO_2_ and Al_2_O_3_/TiO_2_ [165]. These data suggest that most of the previously reported advanced synthesis methods for composite powders still require further scale-up and industrialization. When achieved, this goal will allow high-quality nanocomposite powders to become commercially available, with an expected positive impact on the performance of the produced composite ceramics.

## 5. Conclusions

There are several reasons for which nanocomposite ceramics can present enhanced mechanical properties as respect to monolithic materials. Second-phase particles are very effective in reducing the matrix grain growth during sintering, due to their *pinning* effect. This has a general positive effect on the mechanical properties, particularly on the fracture strength and hardness, as predicted by the Hall-Petch relation and also experimentally observed. The microstructural refinement also leads to flaw size reduction, with a positive impact on strength and reliability of structural composite ceramics. Increments of fracture toughness in nanocomposite ceramics were observed as well, mainly attributed to microcracking, crack deflection and crack bridging toughening mechanisms.

To fully exploit such interesting properties, location, distribution, size and morphology of second-phase grains should be carefully controlled. Inter-granular particles exert an effective *pinning* on the matrix grain size, with beneficial effects on both room and high temperature mechanical properties. On the other hand, intra-type grains (having proper thermal expansion coefficient) may strengthen both matrix/matrix and matrix/second-phase grain boundaries, thus changing the crack path (from inter- to intra-granular fracture mode) and inducing both strengthening and toughening effects. For what concerns the size of the second-phase particles, a key example is provided by the Al_2_O_3_/ZrO_2_ system. To exert the tetragonal-to-monoclinic (*t*-*m*) toughening effect, the tetragonal zirconia grains should have a size ranging between two critical values: a higher critical size, above which spontaneous *t*-*m* transformation spontaneously occurs during cooling, and a lower critical size, below which the transformation is hindered because of excessive stabilization. In addition, a homogeneous distribution of zirconia grains in the Al_2_O_3_ matrix should be achieved, since zirconia clusters could lead to localized ageing phenomena, whereas Al_2_O_3_ aggregates could behave as preferential site for crack propagation.

Achieving an optimal distribution of second-phase particles in a ceramic matrix powder is a still challenging issue, particularly when nanocrystalline particle are used. In fact, due to the extremely high specific surface, nanoparticles have an intrinsically high tendency to agglomeration.

In spite of this, most of the nanocomposite ceramic powders are produced by the traditional mixing and milling method of the constituent oxides, with limitations regarding the distribution of the ultrafine particles in the composite feedstock. A number of advanced methods have been applied to directly synthesize composite nanopowders, such as the vapor-phase reaction synthesis, the combustion synthesis, the wet-chemical methods, the spray decomposition and the solution combustion methods. Several examples of compositions developed by these methods are currently present in literature, but in most of the cases not yet transferred to an industrial production. An emerging technology implies the surface modification of ceramic particles with inorganic precursors of the second-phases. This method can be considered as a compromise between the powder mixing technique and the chemical ones and allow an improved control of the final microstructure. The process implies the use of only inorganic precursors and aqueous media, making this strategy much simpler and potentially transferable to an industrial scale production.
